# Risk factors for prolonged length of hospital stay following elective hip replacement surgery: a retrospective longitudinal observational study

**DOI:** 10.1136/bmjopen-2023-078108

**Published:** 2024-08-21

**Authors:** Rebecca Wilson, Ruta Margelyte, Maria Theresa Redaniel, Emily Eyles, Tim Jones, Chris Penfold, Ashley Blom, Andrew Elliott, Alison Harper, Tim Keen, Martin Pitt, Andrew Judge

**Affiliations:** 1 National Institute for Health and Care Research Applied Research Collaboration West (NIHR ARC West) at University Hospitals Bristol and Weston NHS Foundation Trust, University of Bristol, Bristol, UK; 2 Population Health Sciences, Bristol Medical School, University of Bristol, Bristol, UK; 3 Translational Health Sciences, Bristol Medical School, University of Bristol, Bristol, UK; 4 The University of Sheffield, Sheffield, UK; 5 Southmead Hospital, Bristol, UK; 6 National Institute for Health and Care Research Applied Research Collaboration South-West Peninsula (PenARC), University of Exeter, Exeter, UK; 7 Medical School, University of Exeter, Exeter, UK; 8 North Bristol NHS Trust, Westbury on Trym, Bristol, UK

**Keywords:** Hip, Adult orthopaedics, Hospitalization, Primary Health Care

## Abstract

**Abstract:**

**Objectives:**

Our aim was to identify which patients are likely to stay in hospital longer following total hip replacement surgery.

**Design:**

Longitudinal, observational study used routinely collected data.

**Setting:**

Data were collected from an NHS Trust in South-West England between 2016 and 2019.

**Participants:**

2352 hip replacement patients had complete data and were included in analysis.

**Primary and secondary outcome measures:**

Three measures of length of stay were used: a count measure of number of days spent in hospital, a binary measure of ≤7 days/>7 days in hospital and a binary measure of remaining in hospital when medically fit for discharge.

**Results:**

The mean length of stay was 5.4 days following surgery, with 18% in hospital for more than 7 days, and 11% staying in hospital when medically fit for discharge. Longer hospital stay was associated with older age (OR=1.06, 95% CI 1.05 to 1.08), being female (OR=1.42, 95% CI 1.12 to 1.81) and more comorbidities (OR=3.52, 95% CI 1.45 to 8.55) and shorter length of stay with not having had a recent hospital admission (OR=0.44, 95% CI 0.32 to 0.60). Results were similar for remaining in hospital when medically fit for discharge, with the addition of an association with highest socioeconomic deprivation (OR=2.08, 95% CI 1.37 to 3.16).

**Conclusions:**

Older, female patients with more comorbidities and from more socioeconomically deprived areas are likely to remain in hospital for longer following surgery. This study produced regression models demonstrating consistent results across three measures of prolonged hospital stay following hip replacement surgery. These findings could be used to inform surgery planning and when supporting patient discharge following surgery.

**STRENGTHS AND LIMITATIONS OF THIS STUDY**

- Our sample was relatively large which gave us well-powered models with good discrimination.
- We were able to demonstrate similar results across three different measures of prolonged hospital stay, suggesting our results are consistent across different healthcare metrics.
- The data were collected from one site in England, which may not be generalisable nationally or internationally.
- The data were from pre-COVID-19 and do not reflect the impact the pandemic had on hospital efficiency.
- It is likely that there are unmeasured confounders that may explain some of the associations we found.

## Introduction

 Primary hip replacement is a common elective surgical procedure for the treatment of end stage osteoarthritis^1^ and is a generally safe and successful surgery.^2 3^ In the UK, between 95 000 and 100 000 hip replacement surgeries were performed each year in the 4 years preceding the COVID-19 pandemic (2016–2019); the majority of surgeries being on women (59.9%) and older adults (median age=69 years).^1^

The estimated lifetime risk of hip replacement surgery in the UK is 11.6% for women and 7.1% for men.^4^ The number of hip replacement surgeries performed in the UK has risen consistently since 2005 (with the exception of 2020).^1^ Osteoarthritis in hips in the older population is common and it is anticipated that the number of hip replacement surgeries will rise in the coming years.^5 6^ Hip replacement surgeries are forecast to increase by 38% in the UK by 2060, particularly in older adults.^7^

This growing demand for surgery is likely to increase the burden on health services. Waiting list times for primary hip replacement surgeries were increasing even prior to the pandemic,^8^ with the COVID-19 pandemic leading to a large drop in numbers of surgeries and referrals.^1 8^ The COVID-19 pandemic created such a backlog for hip replacement surgeries that if surgery capacity can be increased by 5% it will take 10 years to clear the backlog, or 5 years if surgeries are increased by 10%.^1^

Longer waiting time for surgery is associated with worse quality of life for patients^9^ and poorer surgical outcomes.^10^ Wait list time is directly related to hospital capacity and patient throughput.^11^ Therefore, waiting lists could be reduced and hospital efficiency improved by minimising the length of time patients stay in hospital post surgery; hospital throughput quickens when beds are available at a faster rate. Identifying which patients are likely to spend longer in hospital following surgery could be used to inform surgery planning and improve hospital throughput.

Length of hospital stay following hip replacement surgery has declined in recent years.^12^,^14^ This has favourable economic implications, and shorter length of stay does not negatively impact patient outcomes and is not associated with major complications or readmission,^14^,^16^ thus benefitting both the healthcare provider and patient.

The aim of this study was to identify the risk factors for prolonged hospital stay following primary hip replacement surgery.

## Methods

### Data sources

This is a longitudinal observational study that uses routinely collected data from an NHS Trust in South-West England. Patients who received elective primary hip replacement surgeries between 2016 and 2019 were identified using a combination of OPCS4 procedure and surgical site codes in the Trust’s electronic health records (EHR) (see online supplemental table S1). Patient characteristics (age, sex, deprivation quintile and comorbidities) and admission-related variables (length of stay and time and date of admission and discharge) were extracted from EHR. Information describing the Trust’s ratio of emergency to elective surgeries, daily emergency (non-elective) admissions and daily non-elective occupied beds was obtained from the Hospital Episode Statistics admitted patient care dataset.^17^ This study was reported in line with the REporting of studies Conducted using Observational Routinely-collected health Data reporting guidelines.^18^

### Outcome variables

Prolonged hospital stay was measured using three variables: (1) a binary measure of prolonged length of stay (0= ≤7 days, 1= >7 days); (2) length of stay in days using a continuous variable with an upper limit of 30 days: patients whose length of stay exceeded 30 days (these were a small number of individuals, 0.9% of all surgeries and were considered outliers) were coded as missing for this variable and (3) a derived binary variable to indicate patients discharged from hospital at a later date than they were declared medically fit for discharge (if no date was recorded when the patient was medically fit for discharge or if the patient was discharged on the same day they were declared medically fit for discharge, this was coded as 0; if date medically fit for discharge preceded discharge date, this was coded as 1).

### Predictive factors

We included patient-related, admission-related and hospital-related factors. Patient-related factors included: a continuous measure of age on admission, patient sex (0=male, 1=female), patient area-level deprivation (IMD quintile derived from LSOA^19^), comorbidities (measured by the weighted Charlson Comorbidity Index^20^) and a categorical measure of time since last all-cause hospital discharge (0–2 months, 2–12 months, 12 months or more or no previous admissions). Admission-related factors included: a categorical measure of admission hour (06.00–12.00, 12.00–18.00, 18.00–06.00), the day of the week, season of admission (winter: December–February; spring: March–May; summer: June–August; autumn: September–November) and year of admission. Hospital-related factors included: the Trust monthly emergency/elective admissions ratio, daily non-elective admissions and daily non-elective occupied beds.

### Statistical analysis

Descriptive statistics were presented for the complete case sample and outcome variables were also tabulated with descriptive variables (proportions were displayed for the two binary outcomes and mean length of stay for the continuous outcome).

We checked the distributions of continuous variables for normality and the linearity of the relationships between risk factors and outcome variables (for linear regression models) and log odds (for logistic regression models). We tested interactions between age and sex, age and comorbidities, age and season, age and deprivation, season and comorbidities, deprivation and comorbidities, season and deprivation. There was insufficient evidence of any of these interactions to justify including them in the models. There was some evidence to suggest the continuous outcome (length of stay) violated the assumptions for the linear regression model (residuals not normally distributed), so robust SEs were estimated using the Huber-White sandwich estimator.^21^ This approach was used rather than log-transformation as it allows for the straightforward interpretation of a difference between the outcome means on the original scale, whereas interpretation of estimates following log-transformation is less intuitive.

Multivariable logistic regression and linear regression including all predictor variables were performed and C-statistics or R^2^ (for the binary and continuous outcomes, respectively) were obtained. Finally, we used multivariable logistic/linear regression, including all predictor variables, with backwards selection at p<0.1 to produce the final models for each outcome. The predictive factors retained in each model were presented using forest plots.

As the categorical time since previous discharge was derived from a continuous measure of days since last discharge, those missing data for the continuous variable were assumed to have not had a previous discharge and were labelled as ‘never’ (had a previous discharge) in the categorical variable. Descriptive statistics were compared between patients who were assumed not to have had a previous discharge and those that did, and the complete case sample and the non-complete case sample (patients with any missing data) were compared with the total sample (online supplemental table S2). No clear differences were observed between these samples (in age, sex, deprivation, ethnicity or comorbidities), and we concluded that using a complete case sample and including those missing data for the days since last discharge did not show evidence of selection bias to the study.

### Patient and public involvement

Patients and the public were consulted to inform the development of the grant, and in the interpretation of initial results. Our patient and public involvement group shared opinions on the prioritisation of particular patient groups (eg, less complex patients during winter and complex patients in the summer) to maximise efficiency for surgery waiting lists and indicated that this would not be controversial, providing they were kept informed and could rely on the information being told to them. If there is evidence to support rescheduling patients in order to improve efficiency, patients and their caregivers could accept surgeries being moved.

## Results

**Figure 1:**
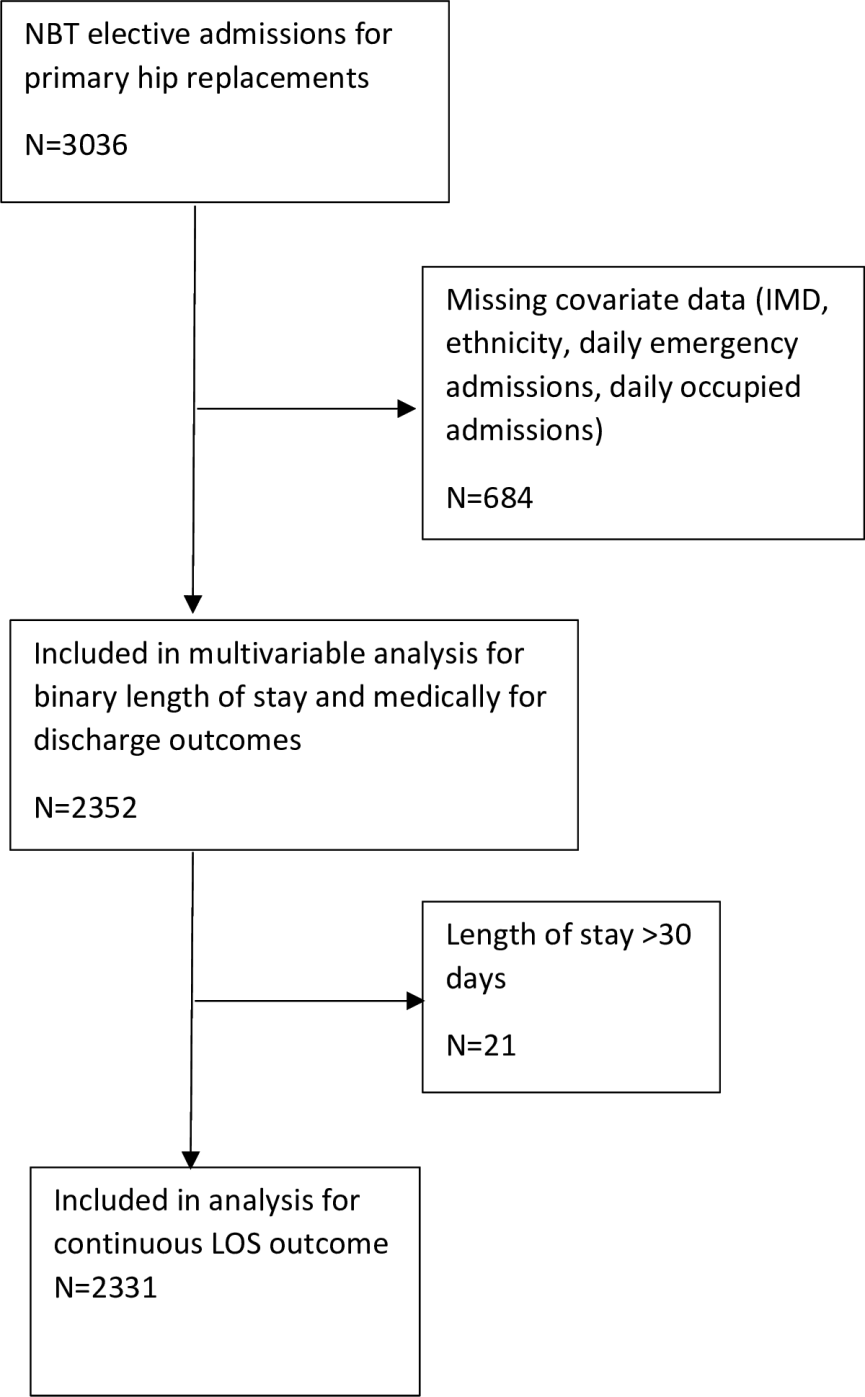
Flow diagram of hip replacement patients included in analysis. LOS, length of stay; NBT, North Bristol Trust

A total of 3036 primary hip replacement surgeries were performed between 2016 and 2019. Of these, 77.5% (n=2352) had complete data for all covariates and were included in the multivariable analysis. 2331 patients (99.1% of complete case sample) had a length of stay of less than 30 days and were included in the linear regression model. (See figure 1.) Participant demographic descriptive statistics are presented in table 1 and full clinical descriptives are included in the supplementary materials (online supplemental table S3).

**Table 1:** Descriptive statistics for elective admissions for primary hip replacements with complete data (n=2352)

Variable	N (%)	Proportion (%) admitted >7 days	Mean length of stay (SD)n=2331	Proportion (%) exceeding medically fit for discharge date
Age at admission				
0–34	60 (2.6)	15.9	3.8 (2.3)	10.32
35–44	93 (4.0)	6.5	4.1 (3.0)	5.4
45–54	233 (9.9)	6.0	4.0 (2.4) (n=232)	4.3
55–64	440 (18.7)	8.6	4.3 (3.0) (n=437)	5.9
65–74	723 (30.7)	14.7	5.1 (4.2) (n=718)	9.5
75–84	650 (27.6)	27.2	6.6 (4.7) (n=644)	13.7
85+	153 (6.5)	51.6	9.6 (5.9) (n=148)	39.9
Age at admission, mean (SD)	67.5 (13.6)	–	–	–
Sex				
Female	1387 (59.0)	20.1	5.1 (3.9) (n=1367)	14.0
Male	965 (41.0)	15.0	4.7 (3.8) (n=956)	7.2
Deprivation (IMD score)				
1 (Least deprived)	679 (28.9)	17.5	5.4 (4.1) (n=674)	9.6
2	551 (23.4)	14.7	5.0 (4.1) (n=547)	7.4
3	409 (17.4)	17.6	5.4 (3.8) (n=406)	11.0
4	381 (16.2)	22.8	6.1 (5.0) (n=377)	16.3
5 (Most deprived)	332 (14.1)	19.6	5.6 (4.6) (n=327)	15.1
Ethnicity				
Non-white	37 (2.5)	–	–	–
Asian	8 (0.6)	–	–	–
Black	15 (1.0)	–	–	–
Mixed	7 (0.5)	–	–	–
Other	7 (0.5)	–	–	–
White	1417 (97.5)	–	–	–
Unknown	898	–	–	–
Comorbidities (weighted Charlson index)				
0	1298 (55.2)	11.7	4.7 (3.6) (n=1290)	8.3
1–2	857 (36.4)	22.8	6.1 (4.6) (n=849)	13.3
3–4	170 (7.2)	37.7	7.6 (5.3) (n=166)	20.5
≥5	27 (1.2)	48.2	8.8 (6.0) (n=26)	25.9
Time since last discharge				
0–2 months	341 (14.5)	31.1	6.9 (5.4) (n=335)	20.8
2–12 months	528 (22.5)	21.4	5.7 (4.5) (n=518)	12.3
12 months or more	354 (15.1)	13.0	5.0 (3.7)	10.5
Never	1129 (48.0)	14.1	5.0 (3.9) (n=1124)	8.0

More than half of patients were aged 65 or over (mean age=67.5, SD=13.6) and 59% were female (table 1). The cohort were representative of the UK cohort undergoing joint replacement of UK patients undergoing joint replacement surgery with over 90% having osteoarthritis. Eighteen per cent of patients (n=424) remained in hospital for more than 7 days; for patients in hospital for 30 days or less, the mean length of stay was 5.4 days (SD=4.3). Medically fit for discharge dates (that preceded discharge dates) were recorded for 11.2% of patients (n=263).

Staying in hospital longer than 7 days was associated with older age, being female, more comorbidities and having a recent hospital admission (compared with at least 2 months ago or never) (table 2). Admissions between 18:00 and 06:00 (compared with 06:00 to 12:00) are associated with length of stay of more than 7 days; however, only 11 patients were included in this group, and thus this was not considered a robust finding.

**Table 2:** Multivariable regression models using backwards selection (at p<0.1)

Variable	Length of stay (admissions >7 days)n=23 48*	Length of stay (<30 days)n=2331	Medically fit for dischargen=2352
	OR (95% CI)	Coef (95% CI)	OR (95% CI)
Age at admission	1.06 (1.05 to 1.08)	0.08 (0.06 to 0.09)	1.06 (1.04 to 1.07)
Sex (female vs male)	1.42 (1.12 to 1.81)	0.63 (0.31 to 0.95)	2.06 (1.52 to 2.79)
IMD score			
1 (Least deprived)	*1.00*	*0.00*	*1.00*
2	0.81 (0.58 to 1.13)	−0.32 (−0.75 to 0.10)	0.76 (0.49 to 1.16)
3	0.88 (0.62 to 1.25)	−0.12 (−0.57 to 0.34)	1.12 (0.73 to 1.71)
4	1.46 (1.04 to 2.05)	0.80 (0.24 to 1.35)	2.11 (1.42 to 3.16)
5 (Most deprived)	1.24 (0.86 to 1.79)	0.32 (−0.24 to 0.87)	2.08 (1.37 to 3.16)
Comorbidities—Charlson index (weighted)			
0	*1.00*	*0.00*	–
1–2	1.84 (1.43 to 2.36)	0.98 (0.61 to 1.34)	–
3–4	2.59 (1.76 to 3.82)	1.74 (0.93 to 2.56)	–
≥5	3.52 (1.45 to 8.55)	2.68 (0.67 to 4.69)	–
Time since last discharge			
0–2 months	*1.00*	*0.00*	*1.00*
2–12 months	0.71 (0.51 to 0.99)	−0.87 (−1.53 to −0.22)	0.56 (0.38 to 0.84)
12 months or more	0.35 (0.23 to 0.52)	−1.59 (−2.25 to −0.93)	0.41 (0.26 to 0.65)
Never	0.44 (0.32 to 0.60)	−1.36 (−1.94 to −0.78)	0.33 (0.23 to 0.47)
Emergency over elective admissions ratio (general/acute, monthly)	–	–	–
NEL admissions, daily (from 01/09/2016)	–	--	1.01 (1.00 to 1.02)
NEL occupied beds, daily (from 01/10/2016)	–	−0.004 (−0.008 to 0.001)	0.99 (0.98 to 0.99)
Admission hour category			
06:00–12:00	*1.00*	*0.00*	*1.00*
12:00–18:00	1.14 (0.69 to 1.86)	0.84 (−0.18 to 1.85)	1.55 (0.90 to 2.66)
18:00-06:00	29.61 (3.29 to 266.66)	5.94 (2.08 to 9.80)	33.00 (7.71 to 141.30)
Year of admission			
2016	–	–	*1.00*
2017	–	–	2.46 (1.30 to 4.67)
2018	–	–	3.13 (1.62 to 6.04)
2019	–	–	2.42 (1.13 to 5.20)
Day of the week of admission			
Sunday	–	–	–
Monday	*1.00*	*0.00*	–
Tuesday	0.82 (0.51 to 1.32)	0.21 (−0.48 to 0.90)	–
Wednesday	0.93 (0.57 to 1.52)	0.19 (−0.56 to 0.93)	–
Thursday	0.62 (0.39 to 1.00)	0.43 (−0.25 to 1.10)	–
Friday	0.95 (0.58 to 1.56)	0.79 (0.05 to 1.53)	–
Saturday	0.20 (0.04 to 0.88)	−1.04 (−1.85 to −0.22)	–
Season of admission			
Winter (December–February)	–	–	–
Spring (March–May)	–	–	–
Summer (June–Aug)	–	–	–
Autumn (September–November)	–	–	–
	C-statistic (95% CI)	R^2^	C-statistic (95% CI)
	0.77 (0.75 to 0.79)	0.154	0.77 (0.74 to 0.80)

* Nn=4 observations omitted from multivariable model (Aadmission day=Sunday dropped from model due to perfect prediction).

Similarly, longer length of stay as measured in days was associated with older age, being female, more comorbidities and having a recent hospital admission (table 2). Again, the spurious association with late admission hour and longer length of stay was observed.

Area-level deprivation, non-elective over elective admissions ratio, number of daily non-elective admissions, non-elective occupied beds, year of admission, admission week day and season of admission were not associated with either the binary or continuous measures of length of stay.

Remaining in hospital when medically fit for discharge was associated with older age, being female, being in the two most deprived quintiles (compared with the least deprived), having a recent hospital admission, a higher number of non-elective daily admissions, fewer non-elective occupied beds and admissions in 2017, 2018 and 2019 (compared with 2016) (table 2). There was also a spurious association with admissions between 18.00 and 06.00. The number of comorbidities, non-elective over elective admissions ratio, admission day and admission season were not associated with remaining in hospital for longer than was medically necessary.

Forest plots displaying all associations for variables retained in the backwards selection models for each outcome measure are provided in the supplementary materials (online supplemental figures S1–S3). Full multivariable models for each outcome variable are provided in the supplementary materials (online supplemental tables S4-S6).

## Discussion

### Summary of results

Prolonged length of hospital stay following primary hip replacement surgery was associated with older age, being female, more comorbidities (although this was not associated with remaining in hospital when medically fit for discharge) and recent hospital admissions. In addition, being medically fit for discharge was also associated with deprivation (specifically, the highest two levels of deprivation) and hospital related factors (daily non-elective admissions and non-elective occupied beds), although the effects were very small. On the whole, the results were consistent across the three outcome measures.

### Comparison of results with previous literature

Our study has corroborated existing evidence, although there is variation in the reported average length of stay, which has reduced over time, by sample characteristics and by country. In a younger cohort (mean age=62 years) from the USA, the mean length of stay was 3.5 days,^22^ and in an older study from the same region of South-West England as our study, the median length of stay was 8 days.^23^ Previous research has cited being female,^23 24^ older age,^1222^,^24^ worse health,^22^,^24^ low socioeconomic deprivation^22^ and day of the week of surgery^24 25^ as predictors of prolonged length of stay following hip replacement surgery. Our results are consistent with previous findings but also add to the evidence base as we used multiple outcome measures of prolonged hospital stay. Recent work by the same authors (unpublished), assessing predictors of prolonged hospital stay following primary knee replacement surgeries in the same NHS Trust, showed largely similar results, with the exception of a consistent association with area-level deprivation and prolonged stay following knee replacement surgery.

### Strengths and limitations

Using linked, routinely collected data provided this study with a relatively large sample which allowed us to develop well-powered, robust statistical models. We used three measures of prolonged hospital stay and observed, on the whole, consistent results across these measures. While there was inevitably missing data, our data checks indicated that this did not bias our data as key characteristics were similar in those with complete and missing data. One limitation of our study is the exclusion of data from 2020 onwards, when the COVID-19 pandemic changed the healthcare landscape, which is not captured in our study. This study was a single-site study which may limit the generalisability of the results to other areas. Furthermore, as an observational study, there are potentially other unmeasured confounders, such as patient’s family or social support, hospital staffing levels or any postoperative adverse events.

Our study produced three robust statistical models demonstrating which patient-related factors were associated with prolonged length of stay following hip replacement surgery. Although the predictive factors from our findings are not modifiable factors which could be used in actively trying to reduce length of hospital stay, it is important to know which patients are at increased risk of longer hospital stays or remaining in hospital longer than is necessary. One approach could be to make targeted improvements to social care; patients at risk of remaining longer in hospital than is necessary need to have an available and safe place to be discharged to, and, in that place, adequate social care and support. This knowledge could also be used to inform service planning. Hospital efficiency could be improved by more informed planning of surgeries, scheduling surgeries for more complex patients (likely to be in hospital for longer) when the hospital is less busy and when more hospital beds are available. Although this approach is sometimes seen as controversial, as it removes the ‘first come first serve’ nature of waiting lists and means some patients could be operated on quicker, it could potentially improve hospital throughput and reduce waiting lists. More research is needed to gain better understanding of the acceptability of this approach to rescheduling surgeries, particularly from the perspectives of clinicians, patients and their families.

## supplementary material

10.1136/bmjopen-2023-078108 online supplemental material 1

## Data Availability

Data may be obtained from a third party and are not publicly available.

## References

[1] Ben-Shlomo Y, Blom A, Boulton C (2021). The national joint registry 18th annual report 2021 [internet]. the national joint registry 18th annual report 2021.

[2] Ferguson R, Prieto-Alhambra D, Peat G (2021). Does pre-existing morbidity influences risks and benefits of total hip replacement for osteoarthritis: A prospective study of 6682 patients from linked national datasets in England. BMJ Open.

[3] Judge A, Cooper C, Williams S (2010). Patient-Reported Outcomes One Year after Primary Hip Replacement in a European Collaborative CohortPRO 1 Yr after Primary Hip Replacement in a European Collaborative Cohort. Arthritis Care Res (Hoboken).

[4] Culliford DJ, Maskell J, Kiran A (2012). The lifetime risk of total hip and knee arthroplasty: results from the UK general practice research database. Osteoarthr Cartil.

[5] Pabinger C, Lothaller H, Portner N (2018). Projections of hip arthroplasty in OECD countries up to 2050. HIP Int.

[6] Culliford D, Maskell J, Judge A (2015). Future projections of total hip and knee arthroplasty in the UK: results from the UK Clinical Practice Research Datalink. Osteoarthr Cartil.

[7] Matharu G, Culliford D, Blom A (2022). Projections for primary hip and knee replacement surgery up to the year 2060: an analysis based on data from The National Joint Registry for England, Wales, Northern Ireland and the Isle of Man. Ann R Coll Surg Engl.

[8] Hampton M, Riley E, Garneti N (2021). THE orthopaedic waiting list crisis TWO SIDES OF THE STORY. Bone Jt Open.

[9] Clement ND, Scott CEH, Murray JRD (2021). The number of patients “worse than death” while waiting for a hip or knee arthroplasty has nearly doubled during the COVID-19 pandemic. Bone Joint J.

[10] Vergara I, Bilbao A, Gonzalez N (2011). Factors and consequences of waiting times for total hip arthroplasty. Clin Orthop Relat Res.

[11] Siciliani L, Hurst J (2005). Tackling excessive waiting times for elective surgery: a comparative analysis of policies in 12 OECD countries. *Health Policy*.

[12] Mealy A, Sorensen J (2020). Effects of an aging population on hospital costs related to elective hip replacements. Public Health (Fairfax).

[13] Molloy IB, Martin BI, Moschetti WE (2017). Effects of the Length of Stay on the Cost of Total Knee and Total Hip Arthroplasty from 2002 to 2013. J Bone Joint Surg Am.

[14] Stambough JB, Nunley RM, Curry MC (2015). Rapid recovery protocols for primary total hip arthroplasty can safely reduce length of stay without increasing readmissions. J Arthroplasty.

[15] Sutton JC, Antoniou J, Epure LM (2016). Hospital Discharge within 2 Days Following Total Hip or Knee Arthroplasty Does Not Increase Major-Complication and Readmission Rates. J Bone Joint Surg Am.

[16] Hunt GR, Crealey G, Murthy BVS (2009). The consequences of early discharge after hip arthroplasty for patient outcomes and health care costs: comparison of three centres with differing durations of stay. Clin Rehabil.

[17] Herbert A, Wijlaars L, Zylbersztejn A (2017). Data Resource Profile: Hospital Episode Statistics Admitted Patient Care (HES APC). Int J Epidemiol.

[18] Benchimol EI, Smeeth L, Guttmann A (2015). The REporting of studies Conducted using Observational Routinely-collected health Data (RECORD) statement. PLoS Med.

[19] Payne R, Abel G (2012). UK Indices of Multiple Deprivation - a Way to Make Comparisons across Constituent Countries.

[20] Charlson M, Szatrowski TP, Peterson J (1994). Validation of a combined comorbidity index. J Clin Epidemiol.

[21] Kirkwood BR, Sterne JA (2010). Essential Medical Statistics.

[22] Inneh IA, Iorio R, Slover JD (2015). Role of Sociodemographic, Co-morbid and Intraoperative Factors in Length of Stay Following Primary Total Hip Arthroplasty. J Arthroplasty.

[23] Foote J, Panchoo K, Blair P (2009). Length of stay following primary total hip replacement. Ann R Coll Surg Engl.

[24] Husted H, Holm G, Jacobsen S (2008). Predictors of length of stay and patient satisfaction after hip and knee replacement surgery: Fast-track experience in 712 patients. Acta Orthop.

[25] Girbino KL, Klika AK, Barsoum WK (2021). Understanding the Main Predictors of Length of Stay After Total Hip Arthroplasty: Patient-Related or Procedure-Related Risk Factors?. J Arthroplasty.

