# Supplementary material for: Risk factors for prolonged length of hospital stay following elective hip replacement surgery: a retrospective longitudinal observational study
Source: BMJ Open. 2024 Aug 21;14(8):e078108. doi: 10.1136/bmjopen-2023-078108 (PMC11340698; doi:10.1136/bmjopen-2023-078108)
Supplement: online supplemental material 1 [file bmjopen-14-8-s001.pdf]

# Supplementary material

Table S1. OPCS-4 codes used to identify primary hip replacement operations

Table S2: Sample characteristics for total sample, those with complete data and those with complete data and with a previous admission

Table S3: Complete table of descriptive statistics for elective admissions for primary hip replacements with complete data

Table S4: Multivariable models for binary length of stay outcome

Table S5: Multivariable models for binary medically fit for discharge date outcome

Table S6: Multivariable models for continuous length of stay outcome

Figure S1: Forest plot of predictors of binary measure of length of stay (>7 days)

Figure S2: Forest plot of predictors of continuous length of stay outcome

Figure S3: Forest plot of predictors of staying in hospital when medically fit for discharge

Supplementary Table S1. OPCS-4 codes used to identify primary hip replacement operations

| Category | Code | Description | Notes |
| --- | --- | --- | --- |
| *Primary Total Hip Replacement* | W37.1 | Primary total prosthetic replacement of hip joint using cement | |
| | W37.8 | Other specified total prosthetic replacement of hip joint using cement | |
| | W37.9 | Unspecified total prosthetic replacement of hip joint using cement | |
| | W38.1 | Primary total prosthetic replacement of hip joint not using cement | |
| | W38.8 | Other specified total prosthetic replacement of hip joint not using cement | |
| | W38.9 | Unspecified total prosthetic replacement of hip joint not using cement | |
| | W39.1 | Primary total prosthetic replacement of hip joint NEC | |
| | W39.8 | Other specified other total prosthetic replacement of hip joint | |
| | W39.9 | Unspecified other total prosthetic replacement of hip joint | |
| | W43.1 | Primary total prosthetic replacement of other joint using cement NEC | |
| | W43.8 | Other specified total prosthetic replacement of other joint using cement NEC | |
| | W43.9 | Unspecified total prosthetic replacement of other joint using cement NEC | |
| | W44.1 | Primary total prosthetic replacement of other joint not using cement NEC | |
| | W44.8 | Other specified total prosthetic replacement of other joint not using cement NEC | |
| | W44.9 | Unspecified total prosthetic replacement of other joint not using cement NEC | |
| | W45.1 | Other primary total prosthetic replacement of other joint NEC | |
| | W45.8 | Other specified total prosthetic replacement of other joint NEC | |
| | W45.9 | Unspecified total prosthetic replacement of other joint NEC | |

| | |
|---|---|
| W52.1 | Primary prosthetic replacement of articulation of bone using cement NEC |
| W52.8 | Other specified prosthetic replacement of articulation of bone using cement NEC |
| W52.9 | Unspecified prosthetic replacement of articulation of bone using cement NEC |
| W53.1 | Primary prosthetic replacement of articulation of bone not using cement NEC |
| W53.8 | Other specified prosthetic replacement of articulation of bone not using cement NEC |
| W53.9 | Unspecified prosthetic replacement of articulation of bone not using cement NEC |
| W54.1 | Primary prosthetic replacement of articulation of bone NEC |
| W54.8 | Other specified prosthetic replacement of articulation of bone NEC |
| W54.9 | Unspecified prosthetic replacement of articulation of bone NEC |
| W93.1 | Primary hybrid prosthetic replacement of hip joint using cemented acetabular component |
| W93.8 | Other specified hybrid prosthetic replacement of hip joint using cemented acetabular component |
| W93.9 | Unspecified hybrid prosthetic replacement of hip joint using cemented acetabular component |
| W94.1 | Primary hybrid prosthetic replacement of hip joint using cemented femoral component |
| W94.8 | Other specified hybrid prosthetic replacement of hip joint using cemented femoral component |
| W94.9 | Unspecified hybrid prosthetic replacement of hip joint using cemented femoral component |
| W95.1 | Primary hybrid prosthetic replacement of hip joint using cement NEC |
| W95.8 | Other specified hybrid prosthetic replacement of hip joint using cement |

| | | | |
|---|---|---|---|
| | W95.9 | Unspecified hybrid prosthetic replacement of hip joint using cement | |
| *Resurfacing / Reconstruction* | W58.1 | Primary resurfacing arthroplasty of joint | Require combination with site + combination codes to ID |
| | W58.8 | Other specified reconstruction of joint | Require combination with site + combination codes to ID |
| | W58.9 | Unspecified other reconstruction of joint | Require combination with site + combination codes to ID |

Supplementary Table S2: Sample characteristics for total sample (N=3036), those with complete data (N=2352), those with complete data and with a previous admission (N=1223) and those with any missing data, ie, non-complete cases (N=1813)

| Main characteristics | | Total sample N=3036 | Complete case sample included in multivariable model with categorical days since last discharge variable N=2352 | Complete case sample included in multivariable model with continuous days since last discharge variable N=1223 | Non-complete cases N=1813 |
|---|---|---|---|---|---|
| Age, mean (SD) | | 67.4 (13.5) | 67.5 (13.6) | 68.2 (13.7) | 66.9 (13.3) |
| Sex, N (%) | Female | 1793 (59.1) | 1387 (59.0) | 735 (60.1) | 1058 (58.4) |
| | Male | 1243 (40.9) | 965 (41.0) | 488 (39.9) | 755 (41.6) |
| IMD score, N (%) | 1 (least deprived) | 867 (28.6) | 679 (28.9) | 371 (30.3) | 496 (27.4) |
| | 2 | 717 (23.6) | 551 (23.4) | 270 (22.1) | 447 (24.7) |
| | 3 | 519 (17.1) | 409 (17.4) | 203 (16.6) | 316 (17.4) |
| | 4 | 484 (15.9) | 381 (16.2) | 206 (16.8) | 278 (15.3) |
| | 5 (most deprived) | 413 (13.6) | 332 (14.1) | 173 (14.2) | 240 (13.2) |
| | (missing) | 36 (1.2) | - | - | 36 (2.0) |
| Ethnicity, N (%) | Non-White | 45 (1.5) | 37 (1.6) | 24 (2.0) | 21 (1.2) |
| | White | 1130 (37.2) | 898 (38.2) | 362 (29.6) | 1024 (56.5) |
| | (missing) | 1861 (61.3) | 1417 (60.3) | 837 (68.4) | 768 (42.4) |
| Comorbidities, N (%) | 0 | 1699 (56.0) | 1298 (55.2) | 605 (49.5) | 1094 (60.3) |
| | 1-2 | 1086 (35.8) | 857 (36.4) | 483 (39.5) | 603 (33.3) |
| | 3-4 | 213 (7.0) | 170 (7.2) | 118 (9.7) | 95 (5.2) |
| | >=5 | 38 (1.3) | 27 (1.2) | 17 (1.4) | 21 (1.2) |

Table S3: Complete table of descriptive statistics for elective admissions for primary hip replacements with complete data (N=2352)

| **Variable** | **N (%)** | **Proportion (%) admitted >7 days** | **Mean length of stay (SD) N=2331** | **Proportion (%) exceeding medically fit for discharge date** |
|---|---|---|---|---|
| **Age at admission** | | | | |
| 0-34 | 60 (2.6) | 15.9 | 3.8 (2.3) | 10.32 |
| 35-44 | 93 (4.0) | 6.5 | 4.1 (3.0) | 5.4 |
| 45-54 | 233 (9.9) | 6.0 | 4.0 (2.4) (N=232) | 4.3 |
| 55-64 | 440 (18.7) | 8.6 | 4.3 (3.0) (N=437) | 5.9 |
| 65-74 | 723 (30.7) | 14.7 | 5.1 (4.2) (N=718) | 9.5 |
| 75-84 | 650 (27.6) | 27.2 | 6.6 (4.7) (N=644) | 13.7 |
| 85+ | 153 (6.5) | 51.6 | 9.6 (5.9) (N=148) | 39.9 |
| Age at admission, mean (SD) | 67.5 (13.6) | - | - | - |
| **Sex** | | | | |
| Female | 1387 (59.0) | 20.1 | 5.1 (3.9) (N=1367) | 14.0 |
| Male | 965 (41.0) | 15.0 | 4.7 (3.8) (N=956) | 7.2 |
| **Deprivation (IMD score)** | | | | |
| 1 (least deprived) | 679 (28.9) | 17.5 | 5.4 (4.1) (N=674) | 9.6 |
| 2 | 551 (23.4) | 14.7 | 5.0 (4.1) (N=547) | 7.4 |
| 3 | 409 (17.4) | 17.6 | 5.4 (3.8) (N=406) | 11.0 |
| 4 | 381 (16.2) | 22.8 | 6.1 (5.0) (N=377) | 16.3 |
| 5 (most deprived) | 332 (14.1) | 19.6 | 5.6 (4.6) (N=327) | 15.1 |

| | | | | |
|---|---|---|---|---|
| **Ethnicity** | | | | |
| Non-White | 37 (2.5) | - | - | - |
| *Asian* | 8 (0.6) | - | - | - |
| *Black* | 15 (1.0) | - | - | - |
| *Mixed* | 7 (0.5) | - | - | - |
| *Other* | 7 (0.5) | - | - | - |
| White | 1417 (97.5) | - | - | - |
| Unknown | 898 | - | - | - |
| **Comorbidities (weighted Charlson index)** | | | | |
| 0 | 1298 (55.2) | 11.7 | 4.7 (3.6) (N=1290) | 8.3 |
| 1-2 | 857 (36.4) | 22.8 | 6.1 (4.6) (N=849) | 13.3 |
| 3-4 | 170 (7.2) | 37.7 | 7.6 (5.3) (N=166) | 20.5 |
| >=5 | 27 (1.2) | 48.2 | 8.8 (6.0) (N=26) | 25.9 |
| **Time since last discharge** | | | | |
| 0-2 months | 341 (14.5) | 31.1 | 6.9 (5.4) (N=335) | 20.8 |
| 2-12 months | 528 (22.5) | 21.4 | 5.7 (4.5) (N=518) | 12.3 |
| 12 months or more | 354 (15.1) | 13.0 | 5.0 (3.7) | 10.5 |
| never | 1129 (48.0) | 14.1 | 5.0 (3.9) (N=1124) | 8.0 |
| **Emergency over elective admissions ratio (general/acute, monthly)**, Mean ± SD | 0.8 ± 0.1 | - | - | - |
| **Emergency admissions, daily**, Mean ± SD | 153.3 ± 21.4 | - | - | - |
| **Emergency occupied beds, daily**, Mean ± SD | 913.3 ± 43.0 | - | - | - |
| **Admission hour category** | | | | |

| | | | | |
|---|---|---|---|---|
| 24.00-06.00 | 2 (0.1) | 100.0 | 28.0 (0)<br>(N=1) | 100.0 |
| 06.00-12.00 | 2217 (94.3) | 17.4 | 5.4 (4.1)<br>(N=2199) | 10.6 |
| 12.00-18.00 | 124 (5.3) | 23.4 | 6.3 (6.1)<br>(N=122) | 16.1 |
| 18.00-24.00 | 9 (0.4) | 88.9 | 10.9 (3.9)<br>(N=9) | 66.7 |
| **Year of Admission** | | | | |
| 2016 | 220 (9.4) | 17.3 | 5.3 (4.8)<br>(N=217) | 7.3 |
| 2017 | 752 (32.0) | 17.6 | 5.5 (4.4)<br>(N=742) | 10.6 |
| 2018 | 730 (31.0) | 19.7 | 5.6 (4.2)<br>(N=725) | 13.7 |
| 2019 | 650 (27.6) | 16.9 | 5.3 (4.2)<br>(N=647) | 10.3 |
| **Month of Admission** | | | | |
| January | 143 (6.1) | 16.1 | 5.3 (4.9) | 9.1 |
| February | 178 (7.6) | 17.4 | 5.4 (4.0)<br>(N=177) | 6.2 |
| March | 196 (8.3) | 21.4 | 5.7 (4.4)<br>(N=191) | 10.2 |
| April | 209 (8.9) | 18.2 | 5.4 (4.5)<br>(N=206) | 12.0 |
| May | 218 (9.3) | 20.6 | 6.1 (4.3) | 16.5 |
| June | 181 (7.7) | 12.2 | 4.6 (2.7)<br>(N=180) | 12.7 |
| July | 195 (8.3) | 23.1 | 6.2 (5.3)<br>(N=193) | 17.4 |
| August | 189 (8.0) | 18.5 | 5.6 (4.6)<br>(N=186) | 8.5 |
| September | 163 (6.9) | 19.6 | 5.4 (4.1)<br>(N=161) | 9.2 |
| October | 238 (10.1) | 18.5 | 5.6 (4.5) | 10.9 |

| | | | | |
|---|---|---|---|---|
| | | | (N=236) | |
| November | 250 (10.6) | 13.6 | 4.8 (3.8) (N=249) | 10.0 |
| December | 192 (8.2) | 17.2 | 5.2 (3.7) (N=191) | 9.9 |
| **Day of the Week of Admission** | | | | |
| Sunday | 4 (0.2) | 100.0 | 12.0 (3.6) | 25.0 |
| Monday | 150 (6.4) | 22.7 | 5.3 (4.3) (N=147) | 10.7 |
| Tuesday | 621 (26.4) | 17.6 | 5.2 (4.2) (N=618) | 11.1 |
| Wednesday | 448 (19.1) | 19.9 | 5.3 (4.6) (N=440) | 11.8 |
| Thursday | 658 (28.0) | 15.8 | 5.7 (4.0) | 10.5 |
| Friday | 408 (17.4) | 20.1 | 5.9 (4.7) (N=402) | 12.5 |
| Saturday | 63 (2.7) | 3.2 | 3.4 (1.6) (N=62) | 6.4 |
| **Season of Admission** | | | | |
| Winter (Dec-Feb) | 513 (21.8) | 17.0 | 5.3 (4.2) (N=511) | 8.4 |
| Spring (Mar-May) | 623 (26.5) | 20.1 | 5.7 (4.4) (N=615) | 13.0 |
| Summer (Jun-Aug) | 565 (24.0) | 18.1 | 5.5 (4.4) (N=559) | 12.9 |
| Autumn (Sep-Nov) | 651 (27.7) | 16.9 | 5.3 (4.2) (N=646) | 10.1 |
| **Spell length of stay**, Mean ± SD | 5.8 ± 6.3 | - | - | - |
| **Spell length of stay up to 30 days**, Mean ± SD **(N=2331)** | 5.4 ± 4.3 | - | - | - |
| **Patients staying >7 days** | | | | |
| <=7 (no) | 1928 (82.0) | - | - | - |

| | | | | |
|---|---|---|---|---|
| >7 (yes) | 424 (18.0) | - | - | - |
| **Patients medically fit for discharge (MFFD)** | | | | |
| with MFFD date before discharge | 263 (11.2) | - | - | - |
| no MFFD date | 2089 (88.8) | - | - | - |
| **Days between MFFD date and Discharge**, Mean ± SD (N=263) | 6.2 ± 8.1 | - | - | - |

Table S4: Multivariable models for binary length of stay outcome (admissions > 7 days)

| Variable | Multivariable logistic regression (no variable selection) N=2348 | | Multivariable logistic regression (backwards selection at p=0.1) N=2348 | |
|---|---|---|---|---|
| | **OR (95%CI)** | **p** | **OR** | **P** |
| **Age at admission** | 1.06 (1.05 to 1.07) | <0.001 | 1.06 (1.05 to 1.08) | <0.001 |
| **Sex (female vs male)** | 1.42 (1.12 to 1.81) | 0.004 | 1.42 (1.12 to 1.81) | 0.004 |
| **IMD score** | | | | |
| 1 (least deprived) | *1.00* | | *1.00* | |
| 2 | 0.80 (0.58 to 1.12) | 0.200 | 0.81 (0.58 to 1.13) | 0.210 |
| 3 | 0.88 (0.62 to 1.25) | 0.467 | 0.88 (0.62 to 1.25) | 0.478 |
| 4 | 1.46 (1.04 to 2.06) | 0.031 | 1.46 (1.04 to 2.05) | 0.031 |
| 5 (most deprived) | 1.23 (0.85 to 1.78) | 0.267 | 1.24 (0.86 to 1.79) | 0.245 |
| **Comorbidities - Charlson index (weighted)** | | | | |
| 0 | *1.00* | | *1.00* | |
| 1-2 | 1.86 (1.45 to 2.39) | <0.001 | 1.84 (1.43 to 2.36) | <0.001 |
| 3-4 | 2.61 (1.77 to 3.86) | <0.001 | 2.59 (1.76 to 3.82) | <0.001 |
| >=5 | 3.79 (1.56 to 9.19) | 0.003 | 3.52 (1.45 to 8.55) | 0.005 |
| **Time since Last Discharge** | | | | |
| 0-2 months | *1.00* | | *1.00* | |
| 2-12 months | 0.72 (0.51 to 1.00) | 0.051 | 0.71 (0.51 to 0.99) | 0.046 |
| 12 months or more | 0.34 (0.23 to 0.52) | <0.001 | 0.35 (0.23 to 0.52) | <0.001 |
| never | 0.44 (0.32 to 0.60) | <0.001 | 0.44 (0.32 to 0.60) | <0.001 |
| **Emergency over elective admissions ratio (general/acute, monthly)** | 0.95 (0.08 to 10.72) | 0.966 | - | |
| **NEL admissions, daily (from 01/09/2016)** | 1.01 (1.00 to 1.02) | 0.237 | - | |
| **NEL occupied beds, daily (from 01/10/2016)** | 0.996 (0.991 to 1.001) | 0.129 | - | |
| **Admission hour category** | | | | |
| 06.00-12.00 | *1.00* | | *1.00* | |
| 12.00-18.00 | 1.15 (0.70 to 1.89) | 0.593 | 1.14 (0.69 to 1.86) | 0.613 |

| | | | | |
|---|---|---|---|---|
| 18.00-06:00 | 32.71 (3.66 to 292.76) | 0.002 | 29.61 (3.29 to 266.66) | 0.003 |
| **Year of Admission** | | | | |
| 2016 | *1.00* | | | |
| 2017 | 1.15 (0.67 to 1.97) | 0.602 | | |
| 2018 | 1.27 (0.69 to 2.32) | 0.445 | | |
| 2019 (until 31st Dec) | 1.00 (0.47 to 2.11) | 0.999 | | |
| **Day of the Week of Admission** | | | | |
| Sunday | - | | - | - |
| Monday | *1.00* | | *1.00* | - |
| Tuesday | 0.80 (0.50 to 1.29) | 0.365 | 0.82 (0.51 to 1.32) | 0.417 |
| Wednesday | 0.91 (0.55 to 1.49) | 0.702 | 0.93 (0.57 to 1.52) | 0.768 |
| Thursday | 0.60 (0.37 to 0.97) | 0.036 | 0.62 (0.39 to 1.00) | 0.050 |
| Friday | 0.87 (0.52 to 1.44) | 0.581 | 0.95 (0.58 to 1.56) | 0.836 |
| Saturday | 0.19 (0.04 to 0.87) | 0.033 | 0.20 (0.04 to 0.88) | 0.033 |
| **Season of Admission** | | | | |
| Winter (Dec-Feb) | *1.00* | | | |
| Spring (Mar-May) | 1.18 (0.82 to 1.70) | 0.363 | | |
| Summer (Jun-Aug) | 0.89 (0.59 to 1.35) | 0.579 | | |
| Autumn (Sep-Nov) | 0.99 (0.67 to 1.46) | 0.971 | | |
| C-statistic | 0.77 (0.75 to 0.79) | | 0.77 (0.75 to 0.79) | |

Table S5: Multivariable models for binary medically fit for discharge date outcome (MFFD date < discharge date)

| Variable | Multivariable logistic regression (no variable selection) N=2352 | | Multivariable logistic regression (backwards selection at p<0.1) N=2352 | |
|---|---|---|---|---|
| | **OR (95%CI)** | **p** | **OR (95%CI)** | **p** |
| **Age at admission** | 1.06 (1.04 to 1.07) | <0.001 | 1.06 (1.04 to 1.07) | <0.001 |
| **Sex (female vs male)** | 2.10 (1.55 to 2.85) | <0.001 | 2.06 (1.52 to 2.79) | <0.001 |
| **IMD score** | | | | |
| 1 (least deprived) | *1.00* | | *1.00* | |
| 2 | 0.75 (0.48 to 1.15) | 0.185 | 0.76 (0.49 to 1.16) | 0.201 |
| 3 | 1.08 (0.70 to 1.66) | 0.736 | 1.12 (0.73 to 1.71) | 0.613 |
| 4 | 2.06 (1.38 to 3.10) | <0.001 | 2.11 (1.42 to 3.16) | <0.001 |
| 5 (most deprived) | 2.00 (1.31 to 3.06) | 0.001 | 2.08 (1.37 to 3.16) | 0.001 |
| **Comorbidities - Charlson index (weighted)** | | | | |
| 0 | *1.00* | | | |
| 1-2 | 1.40 (1.04 to 1.89) | 0.028 | | |
| 3-4 | 1.35 (0.84 to 2.18) | 0.220 | | |
| >=5 | 1.78 (0.64 to 4.97) | 0.268 | | |
| **Time since Last Discharge** | | | | |
| 0-2 months | *1.00* | | *1.00* | |
| 2-12 months | 0.60 (0.40 to 0.89) | 0.011 | 0.56 (0.38 to 0.84) | 0.004 |
| 12 months or more | 0.43 (0.27 to 0.69) | <0.001 | 0.41 (0.26 to 0.65) | <0.001 |
| never | 0.36 (0.25 to 0.52) | <0.001 | 0.33 (0.23 to 0.47) | <0.001 |
| **Emergency over elective admissions ratio (general/acute, monthly)** | 1.86 (0.10 to 33.30) | 0.675 | | |
| **NEL admissions, daily (from 01/09/2016)** | 1.01 (1.00 to 1.02) | 0.143 | 1.01 (1.00 to 1.02) | 0.049 |
| **NEL occupied beds, daily (from 01/10/2016)** | 0.99 (0.98 to 0.99) | <0.001 | 0.99 (0.98 to 0.99) | <0.001 |
| **Admission hour category** | | | | |
| 06.00-12.00 | *1.00* | | *1.00* | |
| 12.00-18.00 | 1.50 (0.86 to 2.63) | 0.152 | 1.55 (0.90 to 2.66) | 0.114 |

| | | | | |
|---|---|---|---|---|
| 18.00-06:00 | 43.42 (7.94 to 237.33) | <0.001 | 33.00 (7.71 to 141.30) | <0.001 |
| **Year of Admission** | | | | |
| 2016 | *1.00* | | *1.00* | |
| 2017 | 2.66 (1.30 to 5.43) | 0.007 | 2.46 (1.30 to 4.67) | 0.006 |
| 2018 | 3.27 (1.49 to 7.14) | 0.003 | 3.13 (1.62 to 6.04) | 0.001 |
| 2019 (until 31st Dec) | 2.55 (1.00 to 6.52) | 0.051 | 2.42 (1.13 to 5.20) | 0.023 |
| **Day of the Week of Admission** | | | | |
| Sunday | | | | |
| Monday | *1.00* | | | |
| Tuesday | 1.25 (0.66 to 2.35) | 0.488 | | |
| Wednesday | 1.24 (0.65 to 2.39) | 0.510 | | |
| Thursday | 1.00 (0.53 to 1.87) | 0.993 | | |
| Friday | 1.16 (0.60 to 2.26) | 0.663 | | |
| Saturday | 0.72 (0.20 to 2.64) | 0.619 | | |
| **Season of Admission** | | | | |
| Winter (Dec-Feb) | *1.00* | | | |
| Spring (Mar-May) | 1.24 (0.79 to 1.94) | 0.358 | | |
| Summer (Jun-Aug) | 0.95 (0.58 to 1.58) | 0.851 | | |
| Autumn (Sep-Nov) | 1.01 (0.63 to 1.64) | 0.952 | | |
| C-statistic | 0.78 (0.75 to 0.81) | | 0.77 (0.74 to 0.80) | |

Table S6: Multivariable models for continuous length of stay outcome (< 30 days) in hip surgeries

| Variable | Multivariable linear regression (no variable selection) N=2331 | | Multivariable linear regression (backwards selection at p<0.1) N=2331 | |
|---|---|---|---|---|
| | **Coef (95%CI)** | **p** | **Coef (95%CI)** | **p** |
| **Age at admission** | 0.08 (0.06 to 0.09) | <0.001 | 0.08 (0.06 to 0.09) | <0.001 |
| **Sex (female vs male)** | 0.63 (0.31 to 0.95) | <0.001 | 0.63 (0.31 to 0.95) | <0.001 |
| **IMD score** | | | | |
| 1 (least deprived) | *0.00* | | *0.00* | |
| 2 | -0.33 (-0.75 to 0.10) | 0.134 | -0.32 (-0.75 to 0.10) | 0.137 |
| 3 | -0.12 (-0.57 to 0.34) | 0.614 | -0.12 (-0.57 to 0.34) | 0.616 |
| 4 | 0.82 (0.26 to 1.38) | 0.004 | 0.80 (0.24 to 1.35) | 0.005 |
| 5 (most deprived) | 0.32 (-0.24 to 0.87) | 0.265 | 0.32 (-0.24 to 0.87) | 0.264 |
| **Comorbidities - Charlson index (weighted)** | | | | |
| 0 | *0.00* | | *0.00* | |
| 1-2 | 0.98 (0.62 to 1.35) | <0.001 | 0.98 (0.61 to 1.34) | <0.001 |
| 3-4 | 1.74 (0.93 to 2.56) | <0.001 | 1.74 (0.93 to 2.56) | <0.001 |
| >=5 | 2.73 (0.71 to 4.75) | 0.008 | 2.68 (0.67 to 4.69) | 0.009 |
| **Time since Last Discharge** | | | | |
| 0-2 months | *0.00* | | *0.00* | |
| 2-12 months | -0.85 (-1.51 to -0.20) | 0.010 | -0.87 (-1.53 to -0.22) | 0.009 |
| 12 months or more | -1.56 (-2.22 to -0.91) | <0.001 | -1.59 (-2.25 to -0.93) | <0.001 |
| never | -1.35 (-1.94 to -0.77) | <0.001 | -1.36 (-1.94 to -0.78) | <0.001 |
| **Emergency over elective admissions ratio (general/acute, monthly)** | -0.67 (-4.01 to 2.67) | 0.693 | - | |
| **NEL admissions, daily (from 01/09/2016)** | 0.01 (-0.01 to 0.02) | 0.228 | - | |
| **NEL occupied beds, daily (from 01/10/2016)** | -0.01 (-0.01 to 0.00) | 0.074 | -0.004 (-0.008 to 0.001) | 0.100 |

| | | | | |
|---|---|---|---|---|
| **Admission hour category** | | | | |
| 06.00-12.00 | *0.00* | | *0.00* | |
| 12.00-18.00 | 0.87 (-0.15 to 1.89) | 0.095 | 0.84 (-0.18 to 1.85) | 0.106 |
| 18.00-06:00 | 6.02 (2.17 to 9.86) | 0.002 | 5.94 (2.08 to 9.80) | 0.003 |
| **Year of Admission** | | | | |
| 2016 | *0.00* | | | |
| 2017 | 0.37 (-0.36 to 1.11) | 0.321 | | |
| 2018 | 0.33 (-0.49 to 1.16) | 0.429 | | |
| 2019 (until 31st Dec) | 0.05 (-0.97 to 1.06) | 0.930 | | |
| **Day of the Week of Admission** | | | | |
| Sunday | | | | |
| Monday | *0.00* | | *0.00* | |
| Tuesday | 0.17 (-0.52 to 0.87) | 0.623 | 0.21 (-0.48 to 0.90) | 0.556 |
| Wednesday | 0.18 (-0.57 to 0.94) | 0.630 | 0.19 (-0.56 to 0.93) | 0.621 |
| Thursday | 0.39 (-0.29 to 1.07) | 0.258 | 0.43 (-0.25 to 1.10) | 0.215 |
| Friday | 0.71 (-0.05 to 1.48) | 0.068 | 0.79 (0.05 to 1.53) | 0.037 |
| Saturday | -0.99 (-1.91 to -0.07) | 0.036 | -1.04 (-1.85 to -0.22) | 0.013 |
| **Season of Admission** | | | | |
| Winter (Dec-Feb) | *0.00* | | | |
| Spring (Mar-May) | 0.22 (-0.29 to 0.72) | 0.392 | | |
| Summer (Jun-Aug) | -0.17 (-0.77 to 0.42) | 0.569 | | |
| Autumn (Sep-Nov) | -0.19 (-0.69 to 0.31) | 0.459 | | |
| $R^2$ | 0.157 | | 0.154 | |

Figure S1: Forest plot of predictors of binary measure of length of stay (>7 days)

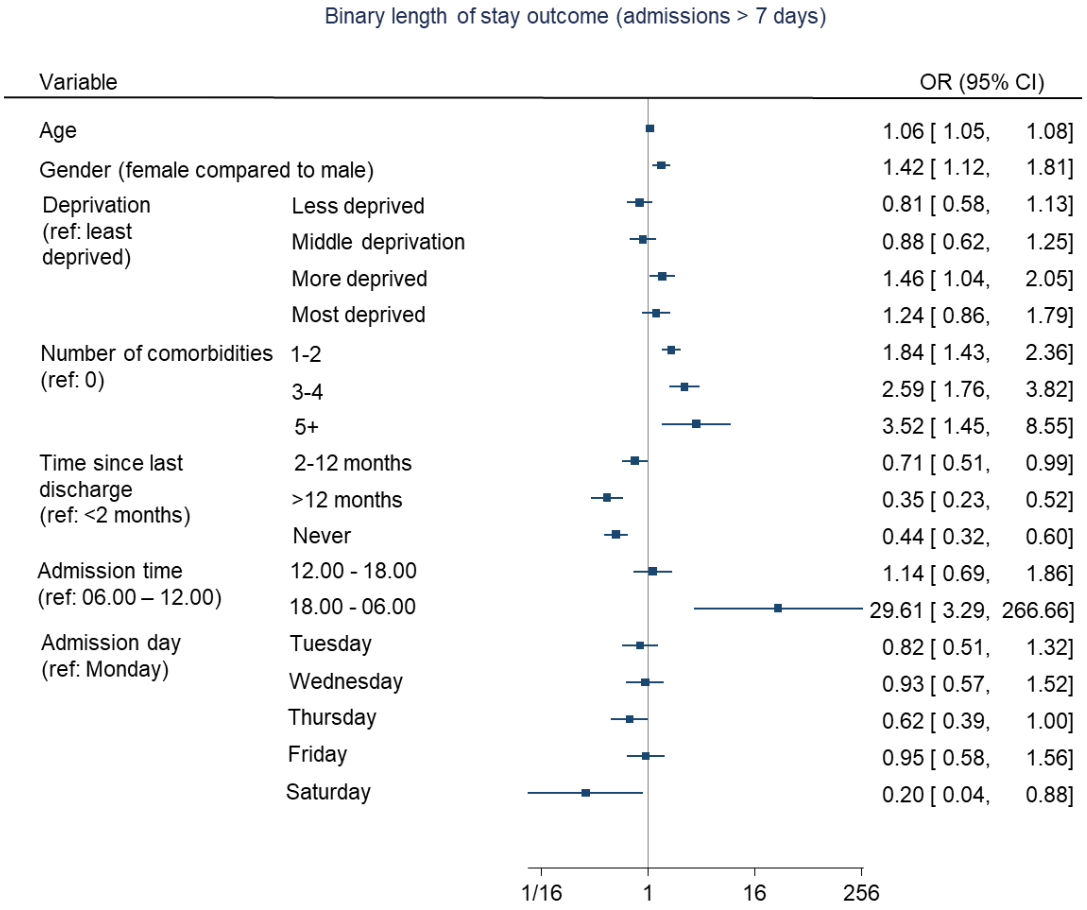


*Figure S1: Predictors of binary measure of length of stay (>7 days)*

Figure S2: Forest plot of predictors of continuous length of stay outcome

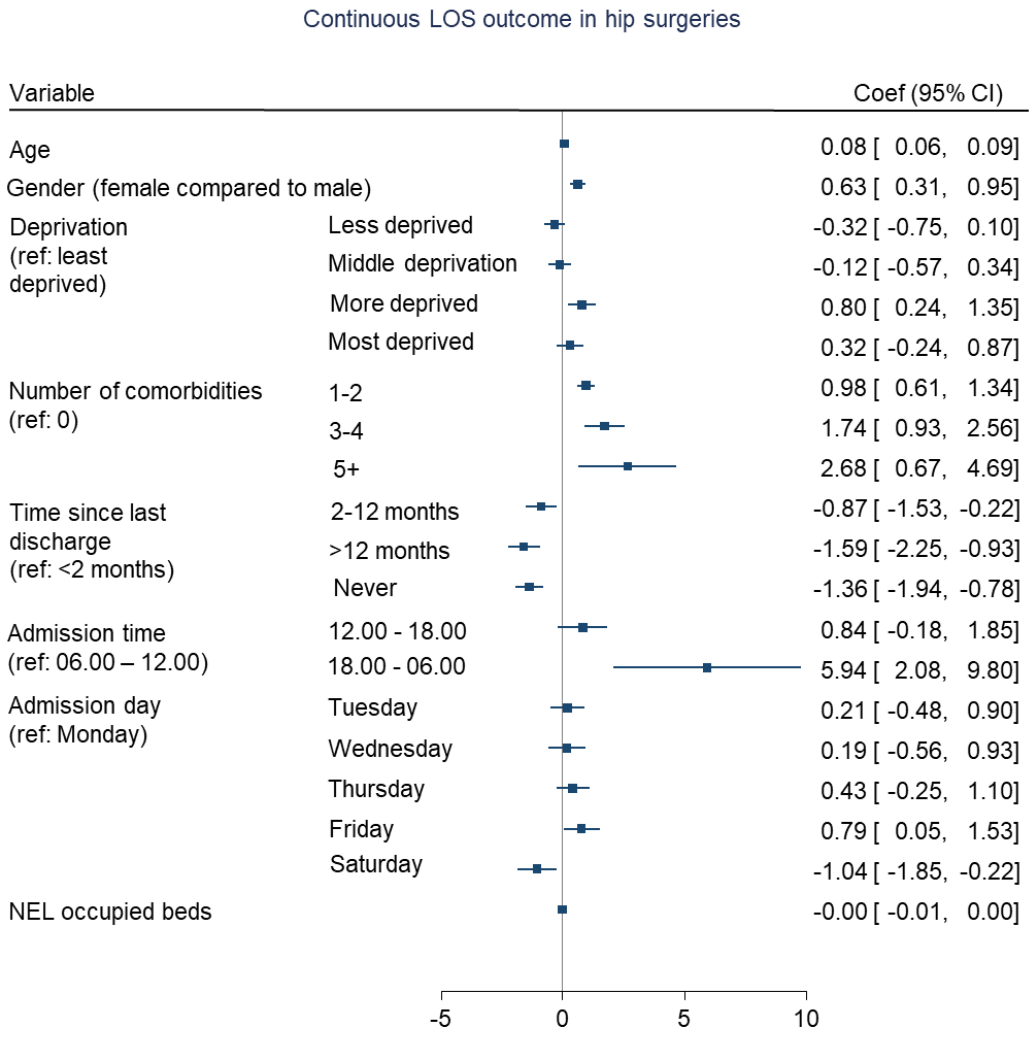


*Figure S2: Predictors of length of stay in days*

Figure S3: Forest plot of predictors of staying in hospital when medically fit for discharge

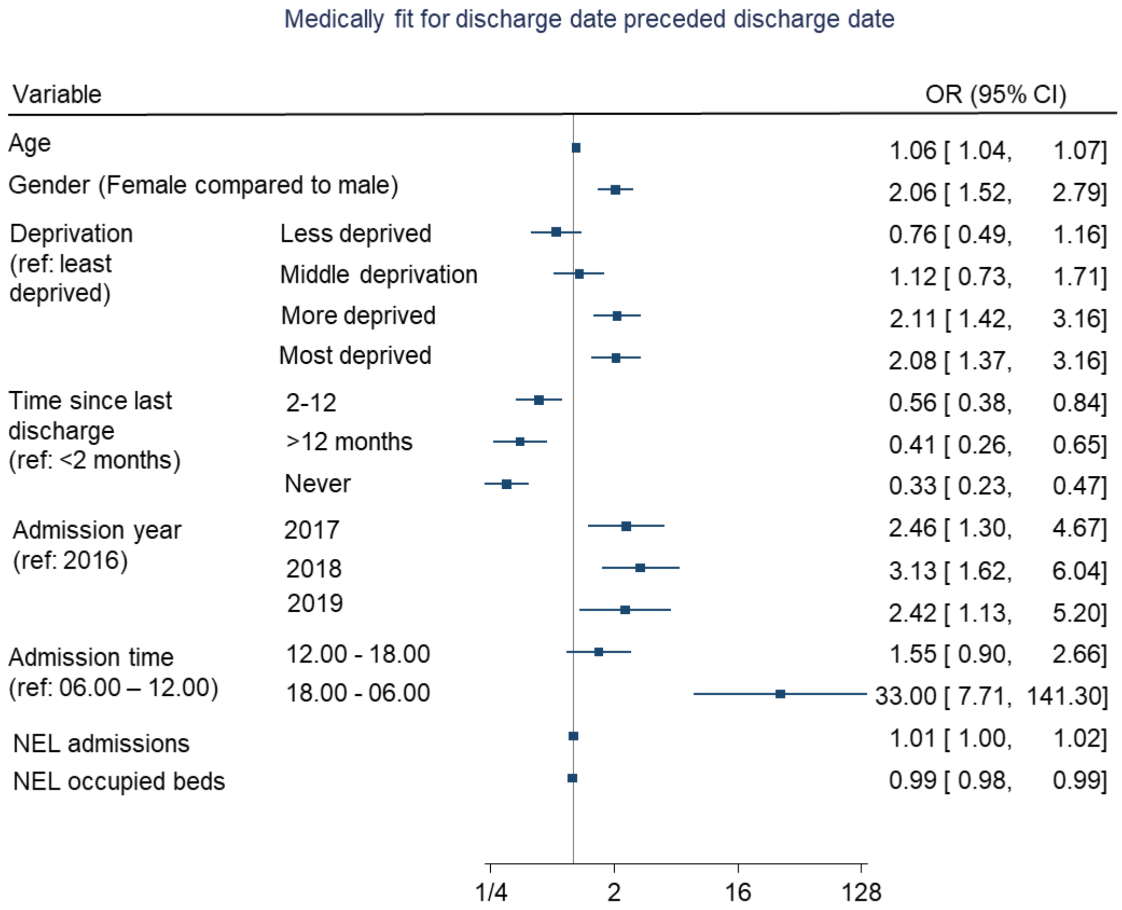


*Figure S3: Predictors of staying in hospital when medically fit for discharge*
