# Supplementary material for: Risk factors for prolonged length of hospital stay following elective hip replacement surgery: a retrospective longitudinal observational study
Source: BMJ Open. 2024 Aug 21;14(8):e078108. doi: 10.1136/bmjopen-2023-078108 (PMC11340698; doi:10.1136/bmjopen-2023-078108)
Supplement: Supplementary file 2 [file bmjopen-2023-078108.reviewer_comments.pdf]

PEER REVIEW HISTORY

BMJ Open publishes all reviews undertaken for accepted manuscripts. Reviewers are asked to complete a checklist review form (**http://bmjopen.bmj.com/site/about/resources/checklist.pdf**) and are provided with free text boxes to elaborate on their assessment. These free text comments are reproduced below.

ARTICLE DETAILS

**TITLE (PROVISIONAL)**	Risk factors for prolonged length of hospital stay following elective hip replacement surgery: a retrospective longitudinal observational study
**AUTHORS**	Wilson, Rebecca; Margelyte, Ruta; Redaniel, Maria Theresa; Eyles, Emily; Jones, Tim; Penfold, Chris; Blom, Ashley; Elliott, Andrew; Harper, Alison; Keen, Tim; Pitt, Martin; Judge, Andrew

VERSION 1 – REVIEW

**REVIEWER**	Hepworth, Graham<br>The University of Melbourne, Statistical Consulting Centre
**REVIEW RETURNED**	07-Feb-2024

**GENERAL COMMENTS**	Being a statistician, I have essentially conducted a statistical review. I don't feel qualified to<br>comment on how novel the research is, whether the outcomes have been chosen optimally,<br>or on the implications. But my impression is that the results could be useful; although they<br>seem to largely confirm what is known already, it is always good to quantify them for use by<br>other researchers or health planners.<br>The paper is well written, clear, and not excessively verbose. The outcomes and predictors<br>are clearly defined. Appropriate caution has been taken with conclusions where not many<br>observations have been involved. The presentation of estimated ORs and associated CIs is<br>standard and appropriate. The use of backwards selection to arrive at a final model, while<br>having disadvantages, is a reasonable method in this situation.<br>I have concerns about some of the statistical methods used in the paper, including in the<br>Supplementary section. Firstly, some of the statistical techniques seem "excess to<br>requirements". In tables S3 to S5, results are presented for three "robust" methods in<br>addition to conventional logistic regression. It's hard to see how including them adds value<br>to the study, other than to confirm that these methods shrink the parameter estimates<br>towards the null (OR = 1). There is also an apparently strange result for Age at admission,

	with estimated ORs above 2, which would result in increased odds of over 7 million in 20 years. Is it on a different scale? Likewise, the calibration plots do not add much to the study. For calibration to be of most use, it should be done with a training data set and a test data set, which requires large numbers of observations, common in machine learning. While the numbers are large here, I'm not sure that it would be very illuminating. Length of stay in hospital is often skewed to the right, and so it is not surprising that it was the case here; a mean of 5.4 and a SD of 4.3 indicates such skewness. In these situations a log transformation is often all that is needed to address the resulting problem of variance heterogeneity in the residuals (which is more important than normality), and such a transformation also avoids having to exclude some observations (as happened here with the 30 day maximum). Using the Huber-White sandwich estimator seems an unnecessary complication. One legitimate reason for avoiding a log transformation is if estimation of the mean rather than the backtransformed mean or median is essential for translation into cost estimates. Such justification should be provided explicitly in the paper if the sandwich estimator is to be persisted with. My final major concern is with the comparison of the complete case sample with the total sample when examining descriptive statistics, this being used to justify using complete cases only. I understand the desirability of using only complete cases (such as not having to use imputation for missing values), and the sample size is certainly adequate. However, it would be better to compare complete cases with non-complete cases to ensure independence. This could be achieved by adding another column to Table S2 for non complete cases, and using that to examine descriptive statistics. Also regarding Table 2, it is not clear why there are only N=1223 cases in the right-hand column. Another statistical concern, which turned out to be inconsequential, was the inclusion of hospital-related predictors in the modelling process without fitting hospital as a random effect in the model (which would have required a mixed model). Assuming a large sample size when there are a limited number of hospitals overstates the significance of such predictors. They were not significant in the analysis anyway, so it didn't affect the reported results, but this was good luck rather than good management. Other comments:

	• Page 5 line 4: “well-performing regression models” It is not clear what this means.<br>• Page 5 line 10: “We were able to demonstrate similar results across three different<br>measures of prolonged hospital stay, suggesting our results are reliable”. These three<br>measures are highly correlated, so this does not provide much evidence of reliability.<br>• Page 5 line 22: “in the three years preceding the COVID-19 pandemic (2016-19)”. Should<br>that be four years?<br>• Page 9 line 16: “We checked the assumptions”. It would be helpful to list which<br>assumptions.<br>• Page 9 line 19: “No interactions were observed”. This is a blanket statement which takes<br>no account of uncertainty, and presumably puts all the weight on hypothesis testing. If a<br>summary statement is to be used, I suggest something like “There was insufficient<br>evidence of any of these interactions to justify including them in the models”.<br>• The number of significant digits used in the tables is generally sensible, but in Table 2,<br>the coefficient for Age at admission and for NEL could do with 2 significant digits.<br>• Page 19 line 10: “being medically fit for discharge was also associated with the highest<br>level of deprivation”. Shouldn’t it be the two highest levels? Also, it is better to say<br>“associated with level of deprivation” (i.e. the factor, not the level) and then describe it.<br>• Page 19 line 12: “the results were consistent across the three outcome measures”. This is<br>hardly surprising, given their correlation.

**REVIEWER**	Kemp, Kyle<br>University of Calgary, Community Health Sciences
**REVIEW RETURNED**	19-Feb-2024

**GENERAL COMMENTS**	Thank you for the opportunity to review this well-written and interesting manuscript, which explored a variety of clinical and demographic risk factors of prolonged hospital stay following elective hip replacement surgery.<br><br>My specific comments are as follows:<br><br>The introduction is excellent and the authors do a nice job of presenting the need for this study within the context of existing research.<br><br>In the methods section, please provide more details about the setting in which the study was conducted. For example, within the Trust, was there a standardized pathway/treatment protocol for hip replacement? This could be a limiting factor, as variation in LOS could be due to differences between hospitals or even clinicians

	within a given site. Also, the availability of wrap-around care (e.g. physical therapy, home care assessment, social worker) may contribute to this. Some of these aspects have been touched upon in the discussion (limitations), but could be bolstered earlier for the reader. Similarly, did the standard of care for patients undergoing hip replacement change over the course of the study period (e.g. early discharge programming being offered).<br><br>Although the work focused upon elective surgery, was the primary reason for surgery captured? Given the finding that younger patient age was associated with longer length of stay, I suspect that these patients were predominantly undergoing surgery due to RA, AVN, or other reasons which may have different treatment course that simple OA.<br><br>I understand that this secondary analysis was only able to capture certain variables. Were any patient-reported outcome measures (PROMS) available/recorded (e.g. WOMAC, HOOS, Harris Hip)? Similarly, were there any objective measures of disability at baseline (e.g. Timed Up and Go test, etc.)?<br><br>The inclusion of time on the wait list may be of benefit to the analyses, if available.<br><br>Similarly, I would advocate for including the day of discharge I the analyses. As an example, tied to my earlier comment, if PT/OT, etc. are not available on weekends, this could prolong the discharge by a day or two to the Monday (thus, skewing the analyses).<br><br>The analyses are excellent, as are the results, Tables, and discussion.<br><br>Thank you again for the opportunity to review this work.

VERSION 1 – AUTHOR RESPONSE

Reviewer 1

Reviewers' comment	Authors' response
I have concerns about some of the statistical methods used in the paper, including in the<br><br>Supplementary section. Firstly, some of the statistical techniques seem "excess to<br><br>requirements". In tables S3 to S5, results are presented for three "robust" methods in	Thank you for your review of our manuscript and for your feedback. We appreciate your view that these analysis are surplus to requirement and have removed the LASSO, elastic net and ridge regression models from the Supplementary Materials (Tables S3, S4 and S5). We have also removed reference to these models in the Methods.

addition to conventional logistic regression. It's hard to see how including them adds value to the study, other than to confirm that these methods shrink the parameter estimates towards the null (OR = 1).	
There is also an apparently strange result for Age at admission, with estimated ORs above 2, which would result in increased odds of over 7 million in 20 years. Is it on a different scale?	We have checked this, and as shown in Table 2, the ORs for Age at Admission are 1.06 (binary length of stay) and 1.06 (medically fit for discharge).
Likewise, the calibration plots do not add much to the study. For calibration to be of most use, it should be done with a training data set and a test data set, which requires large numbers of observations, common in machine learning. While the numbers are large here, I'm not sure that it would be very illuminating.	We have removed the calibration plots from the Supplementary Materials (Figures S4, S5, S6 and S7) and reference to them in the Methods.
Length of stay in hospital is often skewed to the right, and so it is not surprising that it was the case here; a mean of 5.4 and a SD of 4.3 indicates such skewness. In these situations a log transformation is often all that is needed to address the resulting problem of variance heterogeneity in the residuals (which is more important than normality), and such a transformation also avoids having to exclude some observations (as happened here with the 30 day maximum). Using the Huber-White sandwich estimator seems an unnecessary complication. One legitimate reason for avoiding a log transformation is if estimation of the mean rather than the back transformed mean or median is essential for translation into cost estimates. Such justification should be provided explicitly in the paper if the sandwich estimator is to be persisted with.	Thank you for raising this issue. We used the Huber-White estimator to account for the heteroskedasticity in the linear regression model, as described in the Methods. We chose this approach rather than log-transformation as this would complicate the interpretation of the model estimates for the intended audience (clinicians). We have added the following to page 6 line 25 – page 7 line 1, "*This approach was used rather than log-transformation as it allows for the straight-forward interpretation of a difference between the outcome means on the original scale, whereas interpretation of estimates following log-transformation is less intuitive*."
My final major concern is with the comparison of the complete case sample with the total	Thank you for this suggestion. We have added a column to Table S2 reporting the key descriptives for patients with any missing data

sample when examining descriptive statistics, this being used to justify using complete cases only. I understand the desirability of using only complete cases (such as not having to use imputation for missing values), and the sample size is certainly adequate. However, it would be better to compare complete cases with non-complete cases to ensure independence. This could be achieved by adding another column to Table S2 for noncomplete cases, and using that to examine descriptive statistics. Also regarding Table 2, it is not clear why there are only N=1223 cases in the right-hand column.	(non-complete cases) and added this to the methods (page 7 lines 15-16).<br><br>To clarify, the purpose of the column with N=1223 patients, this explored the characteristics of those patients who had a value for the number of days since last discharge (ie, the most complete cases), as those who did not have a value for this continuous variable were coded as 'never' in the categorical days since last discharge variable (see page 7 lines 11-19).
Another statistical concern, which turned out to be inconsequential, was the inclusion of hospital-related predictors in the modelling process without fitting hospital as a random effect in the model (which would have required a mixed model). Assuming a large sample size when there are a limited number of hospitals overstates the significance of such predictors. They were not significant in the analysis anyway, so it didn't affect the reported results, but this was good luck rather than good management.	Thank you for pointing this out.
Page 5 line 4: "well-performing regression models" It is not clear what this means.	We have removed 'well-performing' from the sentence and think following is sufficient, "*This study produced regression models demonstrating consistent results*" (page 3 lines 2-3)
Page 5 line 10: "We were able to demonstrate similar results across three different measures of prolonged hospital stay, suggesting our results are reliable". These three	We have changed this to, "*suggesting our results are consistent across different health care metrics*" (page 3 line 10)

measures are highly correlated, so this does not provide much evidence of reliability	
Page 5 line 22: "in the three years preceding the COVID-19 pandemic (2016-19)". Should that be four years?	Thank you for spotting this mistake, we have changed this to four years (page 3 line 20)
Page 9 line 16: "We checked the assumptions". It would be helpful to list which assumptions	We have added the following to page 6 lines 16-19, "*We checked the assumptions for linear regression (normality of distribution, linearity of the relationship between risk factors and outcome variables, homoscedasticity and statistical independence of errors) and logistic regression (no collinearity between risk factors, linearity of the relationship between the risk factors and the log odds) models*"
Page 9 line 19: "No interactions were observed". This is a blanket statement which takes no account of uncertainty, and presumably puts all the weight on hypothesis testing. If a summary statement is to be used, I suggest something like "There was insufficient evidence of any of these interactions to justify including them in the models".	Thank you for this suggestion, we have added the line "*There was insufficient evidence of any of these interactions to justify including them in the models*" to page 6 line 21-22
The number of significant digits used in the tables is generally sensible, but in Table 2, the coefficient for Age at admission and for NEL could do with 2 significant digits.	We have amended the coefficient for NEL occupied beds (in Table 2) from -0.004 (-0.008 to 0.001) to >-0.01 (>-0.01 to <0.01). All other ratios and coefficients are to 2 decimal places.
Page 19 line 10: "being medically fit for discharge was also associated with the highest level of deprivation". Shouldn't it be the two highest levels? Also, it is better to say "associated with level of deprivation" (i.e. the factor, not the level) and then describe it.	We have changed this sentence to "*medically fit for discharge was also associated with deprivation (specifically, the highest two levels of deprivation)*", page 17, lines 1-2
Page 19 line 12: "the results were consistent across the three outcome measures". This is hardly surprising, given their correlation	We agree that, statistically, these outcomes are correlated, however, our reason for including these three different outcomes is that, clinically, they represent different outcomes for patients, eg, a patient may have a long length of stay in days but they may not have been medically fit for discharge, whereas another patient may have been fit for discharge but remained in hospital, and these patients represent different

	health care channellings, therefore we do think it noteworthy that the predictors are broadly similar.

Reviewer 2

Reviewers' comment	Authors' response
In the methods section, please provide more details about the setting in which the study was conducted. For example, within the Trust, was there a standardized pathway/treatment protocol for hip replacement? This could be a limiting factor, as variation in LOS could be due to differences between hospitals or even clinicians within a given site. Also, the availability of wrap-around care (e.g. physical therapy, home care assessment, social worker) may contribute to this. Some of these aspects have been touched upon in the discussion (limitations), but could be bolstered earlier for the reader. Similarly, did the standard of care for patients undergoing hip replacement change over the course of the study period (e.g. early discharge programming being offered).	Thank you for reviewing our manuscript and for raising several important issues.<br><br>Regarding the possible differences in pathways within the Trust, there is not a standardised pathway with regard to anaesthetic or surgical approach, so there is likely to be as much variation in one hospital as there is across the Trust. This is not something we are able to adjust for in our analyses, but also doesn't give us concern for bias.<br><br>Nurse-led discharge is standard, ie, patients are discharged when nursing and PT staff deem them fit for discharge. Standard of care did not change over the study period.<br><br>With regard to wrap-around care, the majority of patients did not stay in hospital beyond being medically fit for discharge, so a minority of the sample would have been impacted by waiting for social workers or care at home to be in place. NBT Hospital at Home was implemented during this time, but the service saw only three Orthopaedics patients during its initial phase.
Although the work focused upon elective surgery, was the primary reason for surgery captured? Given the finding that younger patient age was associated with longer length of stay, I suspect that these patients were predominantly undergoing surgery due to RA, AVN, or other reasons which may have different treatment course that simple OA.	Thank you for raising this important point. We don't have individual data on primary reasons for surgery, but we do know that the patients are representative of the UK cohort undergoing joint replacement surgery with over 90% having osteoarthritis. We have added this ("*The cohort were representative of the UK cohort undergoing joint replacement of UK patients undergoing joint replacement surgery with over*

	90% having osteoarthritis”) to page 8 lines 10-12.
I understand that this secondary analysis was only able to capture certain variables. Were any patient-reported outcome measures (PROMS) available/recorded (e.g. WOMAC, HOOS, Harris Hip)?<br><br>Similarly, were there any objective measures of disability at baseline (e.g. Timed Up and Go test, etc.)?	In England, the Oxford Hip and Knee Scores are collected nationally pre-operatively and at six months post-op; unfortunately we don't have separate scores for patients undergoing hip replacement surgery.
The inclusion of time on the wait list may be of benefit to the analyses, if available.	Unfortunately we don't have access to individual wait list time data. We only have access to national Rheumatology treatment wait list times, which includes more than just hip surgeries.
Similarly, I would advocate for including the day of discharge I the analyses. As an example, tied to my earlier comment, if PT/OT, etc. are not available on weekends, this could prolong the discharge by a day or two to the Monday (thus, skewing the analyses).	Thank you for suggesting this. Whilst we agree that the weekday is likely to affect timing of discharge, conceptually we don't think it fits in our model. The aim of our study was to identify risk factors for prolonged hospital stay (prior to or at the time of surgery) that could be then used to inform surgery planning. This is why we thought day of admission would be an important variable to consider, as this would be potentially modifiable and could affect length of stay. It would not be feasible to include both day of admission and discharge, as length of stay is a result of these two variables, and this would hinder the model.

VERSION 2 – REVIEW

**REVIEWER**	Hepworth, Graham<br>The University of Melbourne, Statistical Consulting Centre
**REVIEW RETURNED**	05-Apr-2024

**GENERAL COMMENTS**	My concerns have been adequately and methodically addressed. There remain a couple of places where the<br>attempt to address my concern has created more difficulties: The first is the second paragraph of the<br>Statistical analysis section (bottom of page 6). The sweeping statement that a list of assumptions were<br>checked is hard to accept on face value without a brief description of how it was done. For example,<br>“Statistical independence of errors” is primarily a design issue rather than one which can be checked (for

	example) by a graphical display. And “no collinearity between risk factors” has to be an overstatement. It<br>would be better to just mention the key assumptions for which an assessment could be described, such as<br>homoscedasticity (which was already addressed). Using >-0.01 for NEL occupied beds in Table 2 is not very<br>useful. Better to simply use more decimal places so that the number of significant digits is enough (2 would<br>do; 1 is not enough here).

**REVIEWER**	Kemp, Kyle<br>University of Calgary, Community Health Sciences
**REVIEW RETURNED**	13-Apr-2024

**GENERAL COMMENTS**	Thank you to the authors for the time and care that they have taken in addressing my previous comments and suggestions. The responses provided and accompanying edits are thoughtful, and I have have no further questions/comments at this time.<br>Thank you again for the opportunity to review this interesting work.

VERSION 2 – AUTHOR RESPONSE

Reviewer 1

Reviewers’ comment	Authors’ response
…the second paragraph of the Statistical analysis section (bottom of page 6). The sweeping statement that a list of assumptions were checked is hard to accept on face value without a brief description of how it was done. For example, “Statistical independence of errors” is primarily a design issue rather than one which can be checked (for example) by a graphical display. And “no collinearity between risk factors” has to be an overstatement. It<br><br>would be better to just mention the key assumptions for which an assessment could be described, such as homoscedasticity (which was already addressed).	Thank you for highlighting this. We have refined this statement, “We checked the distributions of continuous variables for normality and the linearity of the relationships between risk factors and outcome variables (for linear regression models) and log odds (for logistic regression models). We tested interactions…”
Using >-0.01 for NEL occupied beds in Table 2 is not very useful. Better to simply use more decimal places so that the number of significant digits is enough (2 would do; 1 is not enough here).	The number of decimal places has been increased and this has been changed to *-0.004 (-0.008 to 0.001)*
